# Breakthrough for Anticancer Immunotherapy: Current Advances in Manufacturing Protocols of Chimeric Antigen Receptor-Based Therapies

**DOI:** 10.3390/antib14040105

**Published:** 2025-12-08

**Authors:** Yuxin Qian, Weiwei Ma, Xiao-Ning Xu

**Affiliations:** Department of Infectious Disease, Faculty of Medicine, Imperial College London, London AW7 2AZ, UK; weiwei.ma@hotmail.com

**Keywords:** cancer immunotherapy, chimeric antigen receptor (CAR), cell manufacturing, antibody-derived scFv

## Abstract

Chimeric antigen receptor (CAR)-based immunotherapy has emerged as a transformative strategy in anticancer treatment, driven by advances in CAR construct design, manufacturing platforms, and expansion to diverse immune cell types. The landmark success of CD19-targeted CAR-T cell therapy in B cell malignancies has paved the way for broader clinical applications. As of 2025, the U.S. FDA has approved multiple autologous CAR-T products, underscoring their therapeutic promise. However, challenges persist, including cytokine release syndrome (CRS), neurotoxicity, product inconsistency, and the high cost and complexity of cell manufacturing. Variations in cell source, gene delivery methods, expansion protocols, and CAR design significantly influence the safety, efficacy, and scalability of these therapies. In this review, we comprehensively examine the current advances in manufacturing protocols for CAR-modified T cells, natural killer (NK) cells, and unconventional T cell subsets, including γδ T, invariant natural killer T (iNKT), and mucosal-associated invariant T (MAIT) cells. We also highlight emerging innovations such as in vivo CAR-T generation and off-the-shelf allogeneic approaches. By integrating updated strategies with a critical evaluation of current limitations, this review aims to support the development of standardized, robust, and accessible CAR-based immunotherapies.

## 1. Introduction

For decades, cancer treatment has primarily relied on surgery, radiation, and chemotherapy. However, the advent of immunotherapy has ushered in a new era of precision oncology by harnessing the host immune system to selectively target malignant cells. Among these strategies, adoptive cell therapy, particularly the genetic engineering of T cells to express chimeric antigen receptor (CAR), has emerged as one of the most transformative breakthroughs in hematologic oncology. CARs are synthetic fusion proteins that redirect immune cell specificity toward tumour-associated antigens. They typically consist of an extracellular single-chain variable fragment (scFv) derived from a monoclonal antibody, a hinge/transmembrane domain, and intracellular signaling motifs that activate immune effector functions upon antigen engagement [1]. This modular structure enables CAR-T cells to recognize tumour antigens in an HLA-independent manner and exert potent cytotoxicity.

Clinical studies have demonstrated exceptional efficacy of CAR-T cell therapy, particularly against B cell malignancies. Anti-CD19 CAR-T cells have achieved complete response (CR) rates ranging from approximately 50% to over 90% in patients with relapsed or refractory (r/r) B cell acute lymphoblastic leukemia (B-ALL) and non-Hodgkin lymphomas [2,3]. As of 2025, the U.S. Food and Drug Administration (FDA) has authorized six autologous CAR-T products: four targeting CD19—Kymriah^®^ (Tisagenlecleucel) [4] for r/r ALL, Yescarta^®^ (Axicabtagene Ciloleucel) [5], Breyanzi^®^ (Lisocabtagene Maraleucel) [6] for r/r diffuse large B cell lymphoma (DLBCL), Tecartus^®^ (Brexucabtagene Autoleucel) [7] for r/r mantle cell lymphoma (MCL), alongside two B cell maturation antigen (BCMA)-directed therapies for multiple myeloma (MM), Abecma^®^ (Idecabtagene Vicleucel) [8] and Carvykti^®^ (Ciltacabtagene Autoleucel) [9] (Table 1). All six products have also received marketing authorization from the European Medicines Agency (EMA) [10]. In China, the National Medical Products Administration (NMPA) has approved six CAR-T therapies, including the globally marketed Yescarta^®^ and Carvykti^®^, as well as four domestically developed products, Carteyva^®^ (relmacabtagene autoleucel) [11] for r/r follicular lymphoma (FL), Fucaso^®^ (equecabtagene autoleucel) [12] and Zevor-cel^®^ (zevorcabtagene autoleucel) [13] for r/r MM; and Yorwida^®^ (inaticabtagene autoleucel) for r/r B-ALL [14]. As of June 2025, over 6000 interventional cell therapy trials were registered worldwide, including 1580 CAR-T studies listed on ClinicalTrials.gov [15,16]. It reflects the unprecedented growth of the CAR field and its expanding therapeutic footprint across hematologic cancers and emerging solid tumour indications.

This rapid clinical expansion, however, also highlights the need for innovation to address scalability, cost, and accessibility. While CAR-T therapies have transformed outcomes for many patients, their complex, patient-specific manufacturing and intensive clinical management limit broader application. Variability in T cell quality, activation, transduction efficiency, and expansion kinetics can substantially affect potency and persistence. At the same time, severe toxicities such as cytokine release syndrome (CRS) and immune effector cell-associated neurotoxicity syndrome (ICANS) remain significant clinical concerns [17]. To overcome these barriers, the field is rapidly advancing toward more efficient, standardized, and scalable solutions. Current research focuses on developing allogeneic “off-the-shelf” CAR-T products, exploring alternative immune cell platforms, including γδ T cells, invariant natural killer T (iNKT) cells, mucosal-associated invariant T (MAIT) cells, and natural killer (NK) cells, and integrating automated, closed-system manufacturing technologies. In parallel, in vivo CAR-T generation using viral or non-viral delivery systems is emerging as a transformative approach that could eventually eliminate the need for ex vivo cell manipulation altogether.

In this review, we comprehensively examine the state of the art in CAR-based cell manufacturing, encompassing conventional and unconventional cell sources, gene delivery technologies, expansion platforms, and clinical-grade production systems. We further highlight ongoing innovations aimed at enhancing safety, efficacy, and manufacturing efficiency, setting the stage for the next generation of broadly applicable immunotherapies.

## 2. Manufacturing Protocols for Conventional T Cell Therapy

**Figure 1:**
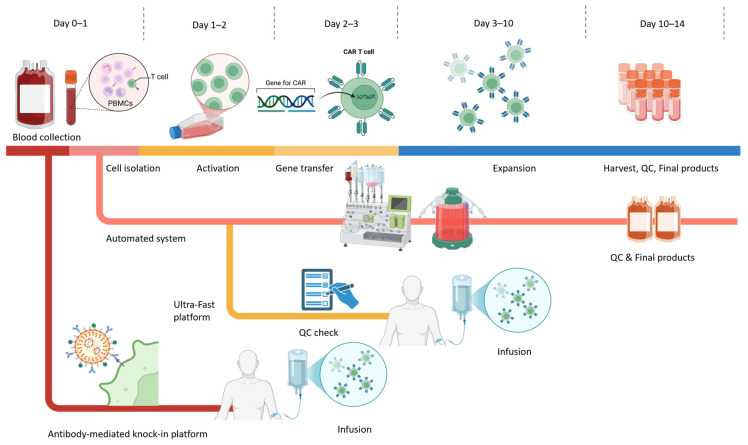
Schematic overview of CAR-T cell manufacturing workflow. The workflow depicts the general ex vivo CAR-T manufacturing process applicable to both autologous and allogeneic settings from Day 0 to Day 14, highlighting conventional processes alongside next-generation rapid and automated platforms. Colours indicate different stages of the CAR-T manufacturing process: collection, activation, gene engineering, expansion, and infusion. Conventional workflows typically involve these steps, resulting in a final product in approximately 10–14 days. In parallel, automated, closed-system platforms integrate activation, transduction, and expansion in a single device to improve standardization, reduce operator variability, and support decentralized GMP manufacturing. Emerging rapid and ultra-fast manufacturing strategies shorten the activation and culture phases, enabling gene transfer as early as Days 1–2 and significantly compressing the overall timeline. Additionally, antibody-mediated knock-in systems (e.g., retargeted lentiviral platforms such as PACK-IT) enable selective T cell gene delivery without the need for prior enrichment or activation and support both ultra-rapid ex vivo production and in vivo CAR-T generation.

The dramatic clinical outcomes of CD19-specific CAR-T cell therapy have boosted research interests in the development of armed immune cells to combat tumours. Minor differences in the manufacturing procedures and the CAR construct design might result in substantial variability in the quality and performance of the final cell products in patients [18,19,20]. The general CAR-T cell production outline includes T cell collection, cultivation, gene transfer and CAR-T cell expansion. The final cell products could be stored through cryopreservation before transfusion to patients, allowing for shipping from manufacturing sites to clinical centres and flexibility in scheduling the adoptive transfer timing (Figure 1).

### 2.1. T Cell Collection and Enrichment

Although the U.S. FDA has approved multiple CAR-T cell products, there is still no universally standardized manufacturing protocol for CAR-T cell therapy. Variability in cell source, processing, and enrichment strategies remains a key determinant of product quality and clinical outcomes. In autologous manufacturing, production typically begins with leukapheresis to isolate white blood cells from the patient’s peripheral blood. Traditionally, lymphocytes were separated using Ficoll density gradient centrifugation [21]. Currently, automated cell-washer machines such as Cytiva^®^ Sepax S-100 (Cytiva, USA), Fresenius Kabi LOVO^®^ (Fresenius Kabi, Germany) and Haemonetics^®^ Cell Saver (Haemonetics Corporation, MA, USA) are used to streamline cell separation, reduce contamination risk, and remove red blood cells, granulocytes, and platelets [21,22]. Approximately 10^6^ to 10^7^ viable T cells are required for downstream CAR modification [23]. Many protocols now incorporate immunomagnetic bead-based selection to enrich specific T cell subsets (e.g., CD4^+^, CD8^+^, or naïve/memory fractions) for improved product consistency. Defined CD4^+^:CD8^+^ ratios, such as the fixed 1:1 ratio described by Turtle et al., have been associated with improved expansion kinetics, persistence, and clinical remission rates in ALL [24]. In certain disease settings, such as chronic lymphocytic leukaemia (CLL), where circulating tumour cells can contaminate leukapheresis products, additional CD3^+^ T cell enrichment may be required to improve manufacturing efficiency [20]. Negative selection is also used to deplete unwanted populations. For example, CD56^+^ bead depletion can remove excessive NK cells (>10%), which may otherwise compete with T cells during culture [25]. The quality of the apheresis product is strongly influenced by patient-related factors, including age, tumour burden, prior therapies, and the hostile tumour microenvironment (TME). For instance, ALL diagnosis and higher platelet count have been linked to lower lymphocyte collection efficiency [26]. Patients heavily pretreated with chemotherapy or radiotherapy often exhibit lymphocytopenia [27], while T cell exhaustion and senescence are common, leading to reduced expansion potential and diminished in vivo persistence [19,28]. These factors must be evaluated at the collection stage to assess the feasibility of CAR-T manufacturing, anticipate expansion challenges, and predict product potency. Standardized apheresis quality assessments and pre-manufacturing fitness assays are increasingly being implemented at clinical sites to optimize product yield and ensure therapeutic-grade cell quality.

### 2.2. T Cell Activation

Robust activation signals are required to initiate T cell proliferation and differentiation during CAR-T cell manufacturing. Several techniques have been developed to mimic physiological T cell activation in vitro, balancing expansion efficiency, phenotype preservation, and transduction competence. The most widely adopted clinical approach remains anti-CD3/CD28 antibody-coated magnetic beads (e.g., Dynabeads^®^ CD3/CD28, Thermo Fisher Scientific, USA), which accounted for the majority of 952 analysed manufacturing protocols in the systematic survey by Vormittag et al. [21]. This strategy offers practical advantages that the antibodies on the bead surface minimize the potential loss during media changes, and the beads can be removed post-expansion via strong magnets. Bead-based stimulation has been shown to induce 10–100-fold higher cytokine production and a less exhausted phenotype compared with OKT3 (anti-CD3) plus IL-2 stimulation [29,30]. Bead-free activator technologies are gaining popularity in recent manufacturing pipelines. Product like Miltenyi’s TransAct™ CD3/CD28 reagent (Miltenyi Biotec, Germany) could provide soluble, biodegradable activation complexes that eliminate bead removal steps, reducing labour, contamination risk, and residual material in the final product [31,32]. Alternative activation systems are being increasingly explored to optimize scalability. Artificial antigen-presenting cells (aAPCs), particularly K562-derived platforms, can deliver sustained and more physiologic activation via engineered expression of co-stimulatory ligands. For example, Shrestha et al. engineered K562-based aAPCs co-expressing CD3, CD28, and CD137L, which supported prolonged expansion in a CAR-dependent manner [33]. Schmidts et al. further optimized this approach by knocking out the low-density lipoprotein receptor (LDLR) to prevent undesired lentiviral transduction, enabling direct co-culture with T cells without additional removal steps. In preclinical ALL models, 2:1 aAPC:T cell co-culture supported robust CAR-T expansion and potent antitumour activity [34]. Activation duration remains a critical process parameter. Stimulation is typically performed for 48 to 72 h, with evidence that shorter ex vivo culture times preserve a less differentiated T cell phenotype, enhanced in vitro effector function, and superior in vivo persistence compared with prolonged culture [35]. Activation extended beyond 5 days has been associated with decreased CD3 expression, reduced viability, lower expansion, and diminished transduction efficiency relative to 3-day activation [36]. Therefore, a balanced activation strategy with appropriate platform and optimized stimulation duration is crucial for generating a CAR-T cell product with high potency, viability, and consistent clinical performance.

### 2.3. CAR Structure and Generations

**Figure 2:**
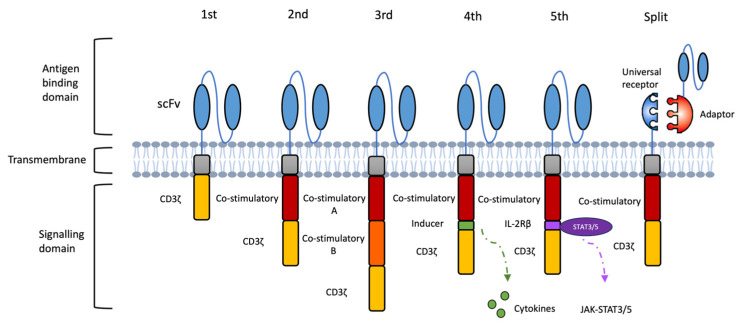
Evolutions of CAR designs. The first-generation CAR consists of an extracellular antigen-binding domain linked to an intracellular CD3ζ signaling module. The second- and third-generation CARs incorporate one or two co-stimulatory domains, respectively, to enhance proliferation, persistence, and anti-tumour activity. Fourth-generation CARs (TRUCKs) are engineered to release immune-modulating molecules, such as proinflammatory cytokines, upon antigen recognition to remodel the tumour microenvironment. Fifth-generation CARs integrate a truncated cytokine receptor domain (e.g., IL-2Rβ) with a STAT3/5-binding motif, enabling antigen-dependent cytokine signaling to further support T cell expansion and function. Novel modular designs, such as split or adaptor CAR systems, separate antigen recognition from signaling by pairing a universal receptor with interchangeable, target-specific adaptors, allowing for adjustable specificity and tuneable activity.

The CAR is embedded in the T cell to determine the specificity and activity of the cell products. A proper CAR construct design is critical for the therapeutic success of CAR-based immunotherapy. Since the introduction of CAR technology, the CAR construct has experienced several generations of renovation, each aimed at overcoming the shortcomings of the previous designs (Figure 2).

First-generation CARs consist of an extracellular scFv for HLA-independent antigen recognition, a transmembrane domain for structural stability, and an intracellular CD3ζ signaling domain for T cell activation and cytotoxic function [20]. Although capable of antigen-specific recognition, these early CD3ζ-based CAR-T cells demonstrated poor in vivo persistence and suboptimal anti-tumour activity due to the absence of co-stimulatory signaling [37,38]. Second-generation CARs incorporate a single co-stimulatory domain, most often CD28 or 4-1BB, alongside CD3ζ, resulting in enhanced proliferation, prolonged survival, and delayed exhaustion [39]. Other co-stimulatory modules, such as OX40, ICOS and CD27, have also been explored to fine-tune T cell persistence and activity in vivo [20]. In patients with r/r B cell malignancies, second-generation CAR-T cells have achieved markedly improved clinical outcomes compared to the first-generation products [40]. Third-generation CARs combine two co-stimulatory domains (e.g., CD28 + 4-1BB or CD28 + OX40) with CD3ζ to deliver stronger and more sustained activation signals. Early-phase clinical studies have shown favourable safety profiles and enhanced anti-tumour efficacy, with more durable responses in certain B-cell lymphoma cohorts [41,42].

The evolution from first- to third-generation CARs was primarily aimed at augmenting T-cell activation, survival, and cytotoxicity. Fourth-generation CARs (TRUCKs) further expanded functionality by enabling T cells to release immune-modulating factors upon antigen encounter, allowing for active reshaping of TME [20]. For instance, CAR-T cells engineered to co-express IL-7 and CCL19 enhanced proliferation, cytotoxicity, and intratumoural recruitment of endogenous immune cells r/r MM (NCT03778346) [43]. Additional cytokines such as IL-12, IL-15, and IL-18 are being incorporated into TRUCK platforms to strengthen T cell function within immunosuppressive niches, although precise control of cytokine release remains essential to prevent systemic toxicity [44]. Fifth-generation CARs build on this concept by introducing truncated cytokine receptor elements (e.g., IL-2Rβ–STAT3/5 motifs) that couple antigen recognition to self-amplifying proliferative and survival signals, potentially reducing reliance on exogenous cytokines [45].

Despite these advances, single-antigen CAR designs remain highly vulnerable to tumour antigen loss or downregulation, one of the most common mechanisms of relapse following CAR-T therapy. Several next-generation CAR architectures have been developed to mitigate antigen escape by broadening tumour coverage. One approach is bispecific CAR format, in which two antigen-binding domains are incorporated into a single receptor. Clinical studies using BCMA/CD19 bispecific CAR-T cells in r/r MM have reported high complete response rates and durable remissions [46]. Beyond fixed dual-target receptors, adaptor-based CAR platforms offer a modular solution to antigen escape by enabling on-demand and reversible retargeting. In the SUPRA CAR system (split, universal, programmable), a universal “zipCAR” receptor on the T cell binds interchangeable “zipFv” adaptors through engineered leucine-zipper interactions, allowing the same CAR-T population to be redirected to new antigens as tumours evolve (Figure 2). This configuration enables rapid switching between antigens using a single cellular product, and competitive zippers can be used as tunable OFF-switches to modulate activity and mitigate toxicity [47]. Similarly, the SpyTag/SpyCatcher platform links a universal CAR scaffold to tumour-specific binders via covalent isopeptide bonds, enabling rapid retargeting, controlled dosing of adaptor molecules, and switching between antigens in response to tumour heterogeneity [48,49].

In addition to antigen escape, next-generation CAR designs increasingly address the toxicity risks inherent to conventional CAR-T therapies, particularly CRS and ICANS. These toxicities arise from excessive cytokine release, myeloid-cell activation, and uncontrolled T cell expansion within the tumour microenvironment. To mitigate these risks, multiple safety-switch mechanisms have been incorporated into CAR constructs. The most clinically advanced is the inducible caspase-9 (iC9) suicide switch, which triggers rapid apoptosis upon administration of a small-molecule dimerizer and has shown effective clinical performance in early trials [50]. Surface elimination markers, such as truncated EGFR (EGFRt), permit antibody-mediated depletion with cetuximab when severe toxicity occurs [51]. More recently, small-molecule, regulated ON/OFF switches, including synthetic Notch receptors, drug-controlled heterodimerization domains, and protease-regulated CARs, have been developed to allow for reversible, dose-dependent control of CAR-T activity [52,53].

These advanced CAR formats represent important developments toward overcoming antigenic heterogeneity and CAR functions. Selecting the appropriate CAR generation and architecture must be tailored to the intended effector cell type, clinical indication, and manufacturing platform to optimise therapeutic potency, durability, and safety.

### 2.4. Gene Delivery

The transfer of the CAR transgene into immune effector cells is a pivotal step in CAR-T cell manufacturing. Current clinical practice relies primarily on viral or non-viral systems to achieve stable CAR expression in patient T cells. Viral vectors remain the most widely used delivery platform, with retroviral and lentiviral systems dominating the field due to their high transduction efficiency, ability to stably integrate the CAR cassette, and safety profile demonstrated in multiple clinical trials [22,54]. For clinical application, all viral vectors must meet stringent GMP standards, necessitating dedicated production facilities and extensive biosafety testing to exclude replication-competent viruses [21]. These requirements contribute significantly to manufacturing costs. Efforts to streamline vector production have included the development of stable packaging cell lines to reduce batch-to-batch variability and cost. For example, Parente-Pereira et al. developed a two-step procedure yielding a fully positive retroviral packaging master cell bank with stable vector expression and high production consistency [23]. In addition, process optimisations such as the use of X-Vivo 15 medium, G-Rex culture vessels for improved nutrient exchange, and RetroNectin-coated surfaces for enhanced viral colocalization with T cells have improved CAR-T yield and transduction efficiency [55].

Alternatives are becoming available to support gene delivery work. In particular, the transposon/transposase system is one of the non-viral gene delivery approaches. Although it is a relatively new approach in the field of cell therapies, it can provide great economic benefits over viral transduction. The transposon/transposase system allows for integration of CAR transgene into the T cell genome with satisfactory efficiency, simple manufacturing procedure and relatively low cost compared to viral transduction. The desired CAR transgene is incorporated into a transposon sequence on the plasmid, encoding the required transposase for later transposon excision and insertion into the T-cell genome. Electroporation is applied to introduce the plasmids to target T cells before activation [56]. Clinical studies using the Sleeping Beauty platform to generate anti-CD19 CAR-T cells in r/r B-cell malignancies have reported durable expression with low risk of insertional mutagenesis [57,58]. Although more trials and research are warranted to confirm the efficacy and safety of this non-viral gene transfer methodology, the early success in the clinical trials has shown its potential as a competent and economical tool for both research and medical use.

### 2.5. T Cell Expansion

Scaling up the cell production to a required infusion level is a challenge in CAR-T manufacturing. Cytokine supplementation plays a central role in shaping the expansion kinetics, composition, and functional phenotype of the final product. The most widely used regimens include interleukin (IL)-2, IL-15, and IL-21, either alone or in combination, to support ex vivo proliferation [20]. However, excessive supplementation with cytokines, especially IL-2 exposure, may lead to a more differentiated and exhausted phenotype of T cells that impairs the killing functions towards cancer cells and reduces the persistence [59]. Comparative studies have shown that supplementation with IL-15 preferentially maintains T cells with a stem cell memory (Tscm)-like phenotype, partly by limiting mTORC1-driven glycolytic metabolism. CAR-T cells expanded with IL-15 alone have demonstrated enhanced proliferative capacity, reduced apoptotic susceptibility, and potent anti-tumour activity in preclinical models [59]. IL-21 has also emerged as a valuable cytokine in expansion protocols. In addition to boosting proliferation, IL-21 can inhibit terminal differentiation into exhausted late-memory phenotypes, thus preserving long-lived, functionally competent T cells [60].

As manufacturing volumes increase, he choice of expansion platform becomes critical. Manual flask cultures are labour-intensive, contamination-prone, and incompatible with large-scale GMP production. Automated, closed-system bioreactors, such as the CliniMACS Prodigy^®^ (Miltenyi Biotec, Germany), Lonza Cocoon^®^ (Lonza, Switzerland), and G-Rex^®^ (Wilson Wolf Manufacturing, USA), provide sterile, tightly controlled environments that minimize operator intervention [22]. These systems have made CAR-T manufacturing more consistent and accessible across clinical centres. Typically, approximately 14 days are required to produce infusion-ready CAR-T products. Comparative studies indicate that CAR-T cells expanded using the CliniMACS Prodigy^®^ system maintain phenotypic and functional equivalence to manually produced products, confirming the feasibility of automated, large-scale production [61,62]. Quality control and in-process monitoring remain essential for GMP compliance, and continued optimization of culture formulations is necessary. Importantly, the successful in-human trial using CliniMACS Prodigy^®^ to generate bispecific anti-CD20/anti-CD19 CAR-T cells (NCT03019055) achieved robust antitumour activity with low toxicity, validating the system’s potential for decentralized, on-site CAR-T manufacturing [63].

Recent technological advances aim to further shortened expansion timelines. Short-cycle manufacturing platforms, such as the PACK-IT (Programmable Antibody-mediated Cellular Knock-In of T cells) system, enable faster CAR-T generation [64]. It uses a mutated vesicular stomatitis virus glycoprotein (VSV-G) envelope that lacks native tropism and is fused with a T cell-specific scFv (e.g., anti-CD3), enabling targeted gene integration directly into T cells. This approach eliminates the need for cell purification or activation and completes ex vivo manufacturing within four days, yielding CAR-T cells with superior potency compared with conventional lentiviral products. Moreover, intravenous administration of anti-CD3 PACK-IT particles in humanized mice successfully generated functional CAR-T cells in vivo, highlighting its potential to unify rapid ex vivo and direct in vivo manufacturing [64].

Building upon these developments, ultra-fast systems such as the Ultra-Fast CAR (UF-CAR) and FasT CAR-T (F-CAR-T) platforms have further compressed production to under 48 h. These one-day protocols optimize activation signals, transduction efficiency, and early-memory phenotype retention to deliver functional CAR-T cells in a single manufacturing cycle [65,66,67]. In a clinical comparison, FasT CAR-T demonstrated encouraging responses and manageable safety in patients with r/r B-ALL while retaining higher proportions of naïve and Tscm-like cells than conventional products [66]. By substantially reducing vein-to-vein time and manufacturing costs, these platforms address logistical delays that can otherwise preclude timely therapy for patients with rapidly progressing malignancies.

Together, these ultra-rapid and programmable systems mark a major step toward next-generation CAR manufacturing, one that is faster, safer, and more compatible with global clinical deployment. Nevertheless, even with the advent of these rapid and automated manufacturing platforms, autologous CAR-T production remains constrained by patient-to-patient variability, high cost, and complex logistics. These ongoing challenges have spurred growing interest in universal, donor-derived allogeneic CAR approaches designed to deliver standardized, readily available therapies.

## 3. From Autologous to Allogeneic: Off-the-Shelf CAR-T Development

**Figure 3:**
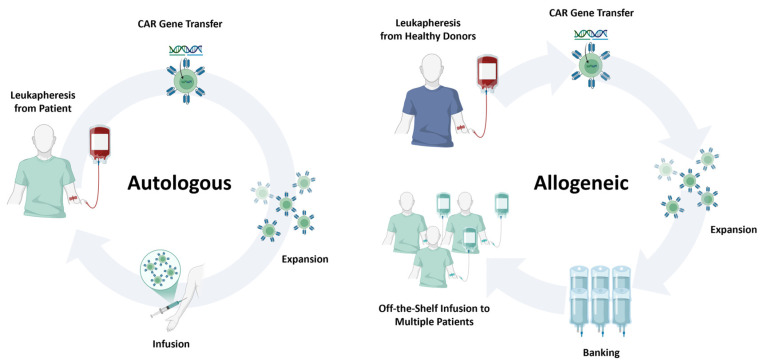
Comparison of autologous and allogeneic CAR cell therapy platforms. Schematic overview highlighting key differences between autologous and allogeneic CAR cell manufacturing. Autologous products are derived from the patient’s own leukapheresis material and reinfused after CAR engineering, offering minimal GVHD risk but prolonged manufacturing times and variable product quality. Allogeneic products originate from healthy donors or engineered cell sources, enabling large-scale, off-the-shelf manufacturing and rapid availability, but requiring gene editing to mitigate alloreactivity and facing potential host immune rejection.

Conventional CAR-T therapies are primarily produced in the autologous setting, in which a patient’s own T cells are collected, genetically modified, expanded, and reinfused. While clinically effective, this personalized approach is costly, time-consuming, and susceptible to production failure, particularly in patients with lymphocytopenia or poor T-cell fitness, resulting in variable product quality. To overcome these challenges, researchers are developing platforms that use healthy donor–derived T cells to create off-the-shelf allogeneic CAR-T products, enabling large-scale manufacture of therapeutic doses from a single donor source. This strategy provides a more economical and readily available alternative to patient-specific therapies. The manufacturing workflow closely parallels that of autologous production but incorporates donor screening to ensure optimal T-cell quality and standardized procedures (Figure 3). By generating multiple doses in advance, allogeneic manufacturing can shorten treatment timelines for patients with urgent clinical needs while reducing overall costs through economies of scale [68,69].

The principal challenge of allogeneic CAR-T therapy lies in preventing graft-versus-host disease (GVHD) and avoiding immune-mediated rejection. The most direct strategy to mitigate GVHD is disruption of the αβ T cell receptor (TCR), as TCR–alloantigen interactions drive alloreactivity [70]. Knockout of either the α or β constant chain prevents functional TCR assembly [71]. Early work by Torikai et al. in 2012 used zinc-finger nucleases to ablate αβ TCR expression, producing anti-CD19 CAR-T cells with preserved anti-tumour activity and no clinically significant GVHD [72]. More recently, CRISPR/Cas9, TALENs, and ARCUS nucleases have been applied to achieve TCR disruption [73,74]. Eyquem et al. demonstrated that targeted CAR insertion into the TCR alpha constant (*TRAC*) locus via CRISPR/Cas9 not only inactivated TCR expression but also improved CAR-T potency and reduced exhaustion in an ALL model [75]. To enhance persistence and resist host immune clearance, additional edits can be introduced. Deletion of CD52 renders CAR-T cells resistant to alemtuzumab-mediated depletion, allowing for selective removal of host CD52^+^ lymphocytes before infusion [76,77]. In the first-in-human trial of αβ TCR– and CD52–deficient allogeneic CD19 CAR-T cells, the product showed a manageable safety profile and preliminary efficacy in r/r LBCL [78]. Alternative immune-conditioning strategies include engineering resistance to nucleoside analogues by disrupting deoxycytidine kinase (dCK), enabling CAR-T cell survival in the presence of purine nucleoside analogue chemotherapy while abrogating host lymphocytes [79].

Building on these foundational studies, CRISPR-based allogeneic CAR-T platforms have progressed rapidly from concept to clinical evaluation, establishing the feasibility of multiplex genome editing for off-the-shelf cell therapies. The CTX110 product from CRISPR Therapeutics employs targeted disruption of *TRAC* and *β2-microglobulin (B2M)* to generate CD19-specific CAR-T cells with reduced GVHD risk and diminished immunogenicity. In the phase I CARBON trial (NCT04035434), CTX110 induced durable remissions in patients with LBCL and a manageable safety profile [80]. The next-generation candidate CTX112 introduces additional edits in *TGFBR2*, *ZC3H12A* to enhance CAR expression, persistence and functional fitness, with preliminary results indicating clinical activity in r/r B cell malignancies (NCT05643742) [81]. Other gene-edited allogeneic CAR-T products, including CB-010 (Caribou Biosciences, USA, NCT04637763) and ALLO-501A (Allogene Therapeutics, USA, NCT04416984), further exemplify how multiplexed genome editing can generate off-the-shelf, TCR-deficient CAR-T cells with reduced host immunogenicity and improved in vivo persistence [82,83]. Across early-phase trials, gene-edited allogeneic CAR-T products have achieved overall response rates of roughly 50–70% in r/r B cell lymphomas, including complete responses in heavily pretreated patients and generally manageable rates of CRS, ICANS, and GVHD [84].

Although still at an early clinical stage, allogeneic CAR-T platforms hold considerable promise as scalable, rapidly deployable cancer immunotherapies. Continued optimisation of gene-editing strategies to mitigate GVHD, evade host rejection, and preserve effector function will be critical for realizing the potential of universal, off-the-shelf CAR-T products.

## 4. Manufacturing Protocols for Unconventional T Cell Therapy

To date, most clinically approved CAR products are based on conventional αβ T cells. While effective, this platform faces several inherent limitations that restrict its broader application. Manufacturing autologous αβ CAR-T cells remains complex and time-consuming, with variability in product quality due to patient-specific factors. Moreover, these cells carry the risk of serious, sometimes life-threatening toxicities, and their activity can be compromised by the diverse human leukocyte antigen (HLA) backgrounds present in genetically heterogeneous populations. From a therapeutic perspective, αβ T cell–based CAR products have demonstrated remarkable efficacy in B cell malignancies but only modest activity in solid tumours [3]. The unfavourable immunosuppressive TME and the heterogeneity of antigen expression in solid cancers present formidable barriers to effective targeting [2]. These challenges highlight the need for next-generation CAR platforms that can overcome antigen escape, function within suppressive tumour niches, and be manufactured efficiently at scale.

**Figure 4:**
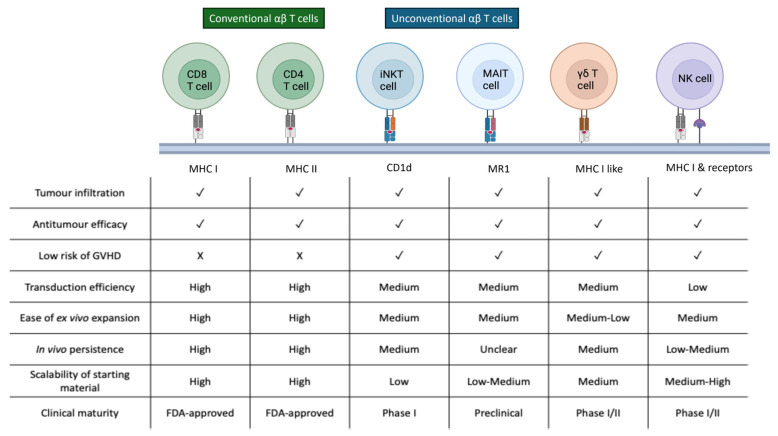
Comparative characteristics of conventional αβ T cells and emerging unconventional immune cell subsets used as platforms for CAR engineering. Key characteristics of conventional αβ T cells and unconventional effector populations, including iNKT, MAIT, γδ T, and NK cells, relevant to CAR-based therapy. These distinctions highlight the unique opportunities and limitations of each platform for autologous and allogeneic CAR product development.

In parallel with optimizing conventional αβ CAR-T cell manufacturing, increasing attention has turned to unconventional T cell subsets, including γδ T cells, iNKT cells, and MAIT cells, as alternative immune effectors. These cell types offer unique biological advantages, such as MHC-independent antigen recognition, intrinsic anti-tumour properties, and potentially lower risks of GVHD, making them attractive candidates for next generation of CAR-based therapies (Figure 4).

### 4.1. Gamma-Delta (γδ) T Cells

γδ T cells are a unique subset of T lymphocytes characterized by their expression of γ and δ T cell receptor chains, in contrast to the conventional αβ T cells. These cells play a critical role in tissue homeostasis and tumour immunosurveillance and have recently gained attention as a promising platform for CAR engineering [85]. The intrinsic properties of γδ T cells offer distinct advantages for cellular immunotherapy, particularly in the context of allogeneic and off-the-shelf CAR-based therapies. One of the most significant features of γδ T cells is their ability to recognize antigens in an MHC-independent manner. This innate-like mode of antigen detection substantially reduces the risk of alloreactivity and GVHD, making donor matching unnecessary and supporting the development of universally compatible cell products [86]. In addition to γδ TCR signaling, these cells respond to a wide array of tumour-associated stress ligands through activating receptors such as Toll-like receptors (TLRs) and natural cytotoxicity receptors (NCRs) [87]. Their non-reliance on classical peptide-HLA complexes expands the scope of targetable tumour antigens and mitigates the impact of tumour antigen heterogeneity and immune escape.

The unique features of γδ T cells highlight their capacity to become a safer and more promising platform of CAR-T therapy in the allogeneic setting. Several groups have developed processing protocols using donor-derived γδ T cells as a CAR backbone to assess their therapeutic potential. Rozenbaum et al. established a robust 14-day manufacturing protocol that begins with peripheral blood mononuclear cells (PBMCs) isolation, followed by stimulation with IL-2 and OKT3 in the presence of zoledronic acid to selectively expand the Vγ9Vδ2 subset. CAR genes were introduced using retroviral transduction, and αβ T cells were removed by magnetic depletion to ensure population purity. Final products exhibited >99% γδ T cell purity and achieved a median 185-fold expansion. These CD19-targeting CAR-γδ T cells demonstrated cytotoxic activity against both CD19-positive and CD19-negative leukaemia cells in murine models, although their anti-tumour potency and persistence were inferior to conventional CAR-T cells, likely due to limited in vivo longevity. Strategies such as repeated infusion or cytokine support may help overcome this limitation [88]. To further optimize γδ T cell manufacturing, Wang et al. explored alternative activation agents including isopentenyl pyrophosphate (IPP) and bromohydrin pyrophosphate (BrHPP), both of which enhanced expansion and lentiviral transduction efficiency. High levels of CAR expression were achieved after 8–10 days of culture, followed by 5 additional days for effective gene transfer. These approaches have laid the foundation for scalable CAR-γδ T cell production [89]. In another example, Capsomidis et al. constructed GD2-specific CARs in donor-derived γδ T cells using a CD28–CD3ζ endodomain. This product demonstrated efficacy in preclinical models of neuroblastoma, highlighting the potential application of γδ CAR-T cells in solid tumours [90]. Given the poor efficacy of conventional CAR-T cells in solid cancers due to the immunosuppressive microenvironment and tumour antigen heterogeneity, the MHC-independent cytotoxicity of γδ T cells could represent a critical advantage.

The clinical translation of CAR-engineered γδ T cells has progressed rapidly in recent years, with several early-phase clinical trials now underway to assess their safety, feasibility, and antitumour potential in both haematologic and solid malignancies (Table 2). The most clinically advanced γδ CAR-T product to date is ADI-001, an allogeneic Vδ1 γδ T cell therapy expressing a CD20-specific CAR. In a phase I trial (NCT04735471), ADI-001 was administered to patients with relapsed or refractory B-cell non-Hodgkin lymphoma and achieved an overall response rate of 75%, with complete remissions in 69% of treated patients, including those who had failed prior CD19-targeted CAR-T therapy [91]. Treatment was well tolerated, with no dose-limiting toxicities, no graft-versus-host disease, and only low-grade cytokine release syndrome observed. Importantly, no TCR gene editing was required to mitigate alloreactivity, owing to the MHC-independent antigen recognition of γδ T cells [91]. A related long-term follow-up study (NCT04911478) is currently monitoring safety and persistence in patients treated with ADI-001 and other γδ CAR-T cell products. Parallel efforts are exploring the use of γδ CAR-T therapies in solid tumours. NCT04107142, a phase I dose-escalation trial sponsored by CytoMed Therapeutics, is evaluating CTM-N2D, an allogeneic Vδ2 γδ T cell product targeting NKG2D ligands, in patients with advanced colorectal, breast, sarcoma, and other refractory solid tumours. Preliminary findings from the first patient cohort indicated good tolerability, enabling dose escalation to the second cohort [92]. In a related approach, Ever Supreme Bio has launched a first-in-human phase I/II study (NCT06150885) evaluating donor-derived Vδ2 γδ T cells transiently co-engineered via mRNA electroporation to express an anti-HLA-G CAR and a bispecific T cell engager targeting PD-L1. This dual-targeting strategy is designed to overcome HLA-G–mediated immune evasion and recruit endogenous CD3^+^ T cells for enhanced tumour clearance. The trial opened in late 2024 for patients with relapsed or refractory solid malignancies; enrolment is ongoing, though clinical outcomes have not yet been reported.

Together, these early studies underscore the feasibility of γδ T cells as an allogeneic backbone for CAR-T therapy. Their innate-like, MHC-independent targeting reduces the risk of GVHD and may facilitate scalable, off-the-shelf production. As clinical evidence accumulates, γδ CAR-T platforms may offer a promising alternative or complement to conventional αβ CAR-T cells, particularly for patients with solid tumours or those ineligible for autologous therapy.

### 4.2. Invariant Natural Killer T (iNKT) Cells

iNKT cells represent a rare subset of innate-like T lymphocytes that express a semi-invariant TCR, composed of a Vα24-Jα18 α-chain paired with a Vβ11 β-chain. In contrast to conventional αβ T cells, iNKT cells recognize glycolipid antigens presented by the non-polymorphic MHC class I-like molecule CD1d [93]. This conserved antigen-presentation pathway, shared across mammals, significantly reduces the risk of alloreactivity and makes iNKT cells inherently safer for use in allogeneic adoptive cell therapies [94]. Functionally, iNKT cells exhibit NK-like cytotoxicity, rapid cytokine release, and tumour infiltration capacity. In preclinical models, they have demonstrated the ability to kill tumour cells directly, modulate myeloid populations, and eliminate tumour-associated macrophages [95,96]. These features, combined with their resistance to GVHD induction and their ability to traffic to inflamed tumour tissues, position iNKT cells as a promising platform for CAR engineering.

Several studies have developed CAR-modified iNKT cells targeting antigens such as CD19, GD2, CD38, and BCMA to explore their therapeutic applicability [97,98,99,100]. Due to their very low natural frequency in human peripheral blood (<0.1%), specialized enrichment protocols are required to enable clinical-scale manufacturing. After PBMC isolation, magnetic bead-based kits targeting the invariant Vα24 TCR are commonly used for iNKT cell selection [96,98]. Activation protocols vary across groups depending on target antigen and expansion goals. Poels et al. utilized IL-2, IL-7, IL-15, and α-galactosylceramide (α-GalCer) to achieve long-term expansion, incorporating 4-1BB signaling to support persistence and reach 300 to 1000-fold expansion within five weeks [100]. Heczey et al. demonstrated that IL-2 and IL-21 in combination with α-GalCer enhanced CD62L expression and iNKT proliferation. Additional OX-40 costimulation further improved antitumour activity [98]. Similarly, Rotolo et al. employed IL-15 and α-GalCer, combined with CD3/CD28 co-stimulation, for robust expansion and CAR transduction [97]. Gene delivery is typically achieved via lentiviral or retroviral vectors over 72 h. Given the rarity of iNKT cells in peripheral blood, restimulation and secondary transduction cycles are often required to reach therapeutic cell numbers [97,98].

In vitro studies have demonstrated that GD2-specific CAR-iNKT cells are capable of killing GD2-positive tumour cells as well as CD1d-expressing tumour-associated macrophages, enhancing tumour control across both direct and indirect mechanisms [99]. The attempts that equipped iNKT cells with CD38-CAR and/or BCMA-CAR also displayed a low risk to cause significant off-tumour toxicity toward normal hematopoietic cells ex vivo [100] and did not induce xenogeneic GVHD responses in murine models [101]. Their Th1-like cytokine profile and CAR-redirected killer functions toward multiple myeloma cell lines highlight their potential as an adoptive immunotherapy strategy [100,101]. In the first-in-human trial (NCT03294954), autologous GD2-targeting CAR-iNKT cells were administered to patients with relapsed or refractory neuroblastoma. The infused cells expanded in vivo, trafficked to bone marrow metastases, and induced signs of tumour regression. No cytokine release syndrome or GVHD was observed, supporting their safety and feasibility [98]. Following these early successes, efforts have turned to allogeneic iNKT cell therapies. A phase I trial (NCT03774654) is currently evaluating healthy donor–derived CD19-directed CAR-iNKT cells engineered with CD28 and IL-15 domains in patients with relapsed or refractory B-cell malignancies. This study aims to assess the persistence, safety, and immune evasion capacity of iNKT cells in an off-the-shelf format. Another trial (NCT05487651), launched in 2022, also investigates allogeneic CD19-CAR-iNKT cells in adult patients with CD19^+^ hematologic malignancies, focusing on dose escalation and treatment tolerability. Together, these trials represent the next phase of translation for iNKT-based CAR therapies and will be instrumental in validating their role as an allogeneic, GVHD-resistant platform for the treatment of lymphoid cancers (Table 2).

### 4.3. Mucosal-Associated Invariant T (MAIT) Cells

MAIT cells are a distinctive subset of innate-like T lymphocytes that express a semi-invariant TCR (Vα7.2-Jα33 with Vβ2 or Vβ13). Unlike conventional αβ T cells, MAIT cells recognize microbial riboflavin metabolites presented by the monomorphic MR1 molecule, rather than polymorphic HLA class I [102,103]. This MR1 restriction fundamentally limits their alloreactive potential and positions MAIT cells as highly suitable for allogeneic, off-the-shelf cellular therapies. A pivotal study by Tourret et al. demonstrated that human MAIT cells are intrinsically non-alloreactive. In both xenogeneic and fully MHC-mismatched allogeneic transplant models, MAIT cells failed to induce GVHD, in stark contrast to conventional αβ T cells [104]. Their inability to recognize alloantigens allows MAIT cells to persist in allogeneic hosts without provoking host-versus-graft or graft-versus-host immune responses, providing a strong biological rationale for developing MAIT cells as a universal donor platform.

MAIT cells are relatively abundant compared with other unconventional lymphocyte subsets. They can comprise up to 10% of circulating T cells and 30–50% of T cells in mucosal organs such as the liver and lung [105]. Their natural tissue residency reflects high expression of homing receptors including CCR6 and CXCR6, enabling efficient trafficking to peripheral tissues and tumours [106,107]. Upon activation, MAIT cells exert potent cytotoxicity through perforin and granzyme release, and secrete pro-inflammatory cytokines such as IFN-γ, TNF-α, IL-17, and IL-2 [108]. They also respond robustly to IL-12, IL-15, and IL-18 in a TCR-independent manner, further amplifying their effector capacity even in settings of limited antigen presentation [102].

The development of MAIT cells as a CAR backbone is still in an early stage, but significant progress has been made in establishing scalable isolation and culture workflows. MR1–5-OP-RU tetramers conjugated to fluorochromes enable highly specific enrichment of MAIT cells and can provide mild TCR stimulation during isolation [109,110]. Flow cytometric sorting based on Vα7.2 and CD161 co-expression remains another reliable approach for obtaining highly pure populations. Standard CD3/CD28 bead stimulation is compatible with MAIT expansion, but cytokine-enriched regimens can yield superior proliferation. Parrot et al. demonstrated over 300-fold expansion using IL-2 and irradiated autologous feeder PBMCs at a 1:10 ratio, providing sufficient output for downstream engineering [109]. Recent preclinical studies have confirmed the feasibility of generating CAR-modified MAIT cells. Lentiviral transduction following MR1-ligand stimulation and cytokine support (IL-2, IL-7, IL-15) enabled stable CAR expression targeting CD19, HER2, or mesothelin [111,112]. CAR-MAIT cells showed potent cytotoxicity comparable to conventional CAR-T cells while producing lower levels of inflammatory cytokines, suggesting a potentially improved safety profile [111]. Notably, MAIT cells could also target MR1-expressing tumour-associated macrophages, permitting dual mechanisms of tumour control and improved performance in immunosuppressive organoid models [112].

Although clinical trials of CAR-MAIT cells have not yet begun as of 2025, translational interest is accelerating. A small but growing patent landscape describes MAIT-cell purification methods, MR1-tetramer applications, and genetic engineering strategies for CAR or TCR modification. These filings remain early and largely academic, but they reflect increased recognition of MAIT cells as a promising allogeneic platform. Given their absence of alloreactivity, natural tissue tropism, dual innate–CAR killing capacity, and resilience within immunosuppressive microenvironments, MAIT cells represent a compelling foundation for next-generation universal cellular therapies. Rigorous preclinical evaluation, optimization of culture and gene-delivery methods, and standardized potency assays will be essential to advance CAR-MAIT cells toward future clinical translation.

## 5. Manufacturing Protocols for Natural Killer (NK) Cell Therapy

NK cells are cytotoxic innate lymphocytes that contribute to tumour immune surveillance by directly targeting transformed or infected cells without prior sensitization. Their function is regulated by a dynamic balance of activating and inhibitory receptors, rather than antigen-specific recognition. NK cell infiltration into tumours has been associated with improved clinical outcomes in several cancers, including lung carcinoma, melanoma, and hepatocellular carcinoma [113,114]. Given their favourable immunobiology and safety profile, NK cells have emerged as an attractive platform for CAR engineering.

Compared to T cells, CAR-NK cells offer several advantages. They are naturally alloreactive-tolerant and do not require strict HLA matching, enabling their use in off-the-shelf settings without inducing GVHD [115]. Furthermore, their limited production of pro-inflammatory cytokines, such as IL-6 and TNF-α, reduces the risk of CRS and neurotoxicity, two major toxicities observed in CAR-T therapy [18,116]. Importantly, NK cells retain intrinsic cytotoxic mechanisms, which allows them to eliminate tumour cells independently of CAR engagement, thereby mitigating the risk of tumour antigen escape [115].

Multiple NK cell sources have been exploited for CAR engineering, including peripheral blood, umbilical cord blood, induced pluripotent stem cells (iPSCs), and the NK-92 cell line [117]. Peripheral and cord blood NK cells are immediately accessible but exhibit donor-to-donor variability and limited proliferative capacity. By contrast, iPSCs provide a renewable, genetically defined, and highly standardized starting population. CAR insertion can be performed at the pluripotent stage, followed by directed differentiation into NK cells under GMP conditions, yielding uniform batches with consistent phenotype and function [118,119]. This top-down manufacturing architecture enables long-term master cell banking and supports true off-the-shelf scalability. Recent iPSC-derived platform, FT596, integrates a CD19 CAR, enhanced CD16 receptor, and IL-15 receptor fusion, showed excellent tolerability with no GVHD and low rates of CRS/ICANS in a phase I trial (NCT04245722) while achieving durable responses in r/r B cell lymphomas [120]. Similar iPSC-based frameworks are now being evaluated for CAR-T generation, offering a theoretically inexhaustible supply of rejuvenated T cells with reduced donor heterogeneity [121]. The NK-92 cell line represents another widely utilized source due to its ease of expansion, high cytotoxic potential, and efficient gene transfer. Clinical studies evaluating NK-92 or CAR-NK-92 products, including HER2-CAR-NK-92 for glioblastoma (NCT03383978), have demonstrated favourable safety profiles without GVHD or high-grade CRS [122,123]. However, because NK-92 cells originate from a malignant clone, they require irradiation prior to infusion to prevent engraftment, which limits persistence and restricts their use predominantly to solid tumour indications or repeated-dose regimens [124,125]. These characteristics distinguish NK-92 from primary and iPSC-derived CAR-NK products and highlight important considerations for platform selection.

Ex vivo NK cell expansion is typically supported by cytokine stimulation with IL-2, IL-15, or IL-21, often in combination with irradiated feeder cells or artificial antigen-presenting cells. Engineered K562 feeder cells expressing membrane-bound IL-15 and IL-21 can dramatically enhance expansion efficiency and cytotoxicity in both primary and iPSC-derived NK cells [126,127]. A major bottleneck in CAR engineering is the intrinsic resistance of NK cells to viral transduction. Conventional retroviral methods achieve only modest efficiencies (22–66%) [117,119]. In contrast, lentiviral vectors pseudotyped with baboon endogenous retrovirus (BaEV) envelope proteins markedly improve gene delivery, enabling transduction efficiencies exceeding 80% in some platforms [127].

To enhance function in immunosuppressive TME, several groups have applied targeted gene editing to improve NK-cell resilience. Deletion of *PD-1* or *CISH* using CRISPR/Cas9 enhances cytotoxicity, metabolic fitness, and in vivo persistence [128,129]. Similarly, knockout of *TGFβR2* confers resistance to TGF-β–mediated suppression without impairing cytolytic activity [130,131]. These modifications aim to equip CAR-NK cells with enhanced resilience against immunosuppressive signals and are being incorporated into next-generation constructs.

Scalable manufacturing is also advancing through automated, closed-system bioreactors. The CliniMACS Prodigy^®^ platform has been adapted for NK-cell processing, enabling GMP-compliant generation of CAR-NK cells with minimal hands-on time. Albinger et al. produced functional CD33-CAR-NK cells using this system, achieving cytotoxicity comparable to manually manufactured batches in preclinical models [132]. Automation is likely to be particularly impactful for iPSC-derived platforms, where large, uniform cell banks are well aligned with fully closed manufacturing workflows. Clinical translation continues to accelerate (Table 2). One of the earliest and most prominent trials (NCT03056339) evaluated UCB-derived CD19-targeted CAR-NK cells engineered to co-express IL-15 and a safety switch, the results demonstrated encouraging remission rates with minimal toxicity [133]. Additional ongoing phase I/II studies, including NCT04847466 and NCT05410717, are evaluating emerging CAR-NK constructs targeting antigens such as Claudin-6, GPC3, mesothelin, and AXL, as well as innovative design strategies for the treatment of solid tumours [134].

Taken together, the favourable immunobiology of NK cells, combined with engineering flexibility, scalable iPSC-based manufacturing, and encouraging early clinical results, highlights CAR-NK therapy as a promising and increasingly mature alternative to CAR-T platforms in adoptive immunotherapy.

## 6. In Vivo CAR-T Cell Therapy

The clinical and logistical challenges inherent to conventional ex vivo CAR-T cell manufacturing have driven the development of in vivo CAR-T strategies. Instead of isolating, activating, and genetically modifying patient T cells outside the body, this approach delivers CAR-encoding constructs directly to endogenous T cells, enabling their reprogramming within the patient. In vivo CAR-T strategies offer the potential to eliminate patient-specific production lines, accelerate treatment timelines from weeks to days, and expand access to cellular immunotherapy beyond specialized manufacturing centres [135]. In vivo CAR-T therapy requires efficient, targeted delivery of CAR-encoding genes to peripheral T cells, and several delivery technologies are under active investigation (Table 3).

### 6.1. Lipid Nanoparticle (LNP)

Among non-viral systems, lipid nanoparticles (LNPs) have emerged as the leading platform for in vivo CAR-T generation due to their clinical precedent in mRNA vaccines, high biocompatibility, and scalable manufacturing. LNPs consist of ionizable lipids, cholesterol, phospholipids, and PEG–lipids and can be conjugated with antibodies or scFvs that recognize T-cell surface markers such as CD3, CD4, CD5, CD7, or CD8. After intravenous administration, these particles are taken up by T cells and release their cargo into the cytoplasm following endosomal escape [136,137]. This transient CAR expression profile without genomic integration provides an excellent safety margin and supports repeated dosing for controlled therapeutic exposure. However, it also limits persistence, necessitating redosing to maintain efficacy. Moreover, innate immune activation through TLR7/8 or RIG-I signaling and hepatic uptake can restrict systemic delivery efficiency [137]. Optimization of lipid composition and targeting ligands is therefore critical to achieve selective, sustained T cell transfection in humans.

The feasibility of this approach has been evaluated in multiple early studies. In a preclinical model, CD3-targeted LNPs carrying CD19-CAR mRNA and IL-6 short hairpin RNA achieved efficient in vivo T-cell transfection, resulting in sustained CAR expression for up to 90 days, potent B cell depletion, and attenuated CRS-associated inflammation [138]. Billingsley et al. optimized ionizable LNP chemistry and achieved extra-hepatic targeting of T cells in vivo, leading to up to 90% B cell depletion [139]. A more advanced mRNA-LNP system to date is the L829 targeted LNP (tLNP) described by Hunter et al. This formulation delivered CAR mRNA directly to CD8^+^ T cells in humanized mice and non-human primates while minimizing hepatic accumulation. The therapy induced rapid and profound B cell depletion, followed by the repopulation of predominantly naïve B cells, reflecting an effective immune system reset. The treatment was well tolerated and caused only mild, transient cytokine elevations [136].

Recently, Bimbo et al. expanded this technology to a DNA-based targeted LNP system termed NCtx, which carries minicircle DNA and transposase mRNA instead of only CAR-mRNA. The particles were dual-targeted to CD3 and CD7, allowing for uptake by both resting and activated T cells and eliminating the need for prior stimulation. This design enabled stable genomic integration of the CAR construct, generating long-lived CAR-T cells directly in the host. In humanized mouse models, a single injection of NCtx-CD19 led to complete leukemia regression and durable survival. Moreover, dual-specific constructs encoding CD19/CD22 CARs maintained CAR expression for more than 40 days [140].

LNP-mediated delivery systems offer a compelling balance of precision, safety, and manufacturability. Transient mRNA formulations minimize genotoxic risk and enable repeat dosing, whereas DNA-based or transposase-assisted designs can extend expression durability. Ongoing refinements in lipid chemistry, targeting ligands, and nucleic acid payload engineering are rapidly advancing these platforms toward clinically viable in vivo CAR-T manufacturing.

### 6.2. Polymeric Nanoparticle

Polymeric and hybrid lipid–polymer nanoparticles provide an alternative non-viral route for in vivo CAR delivery, particularly for DNA-based payloads designed to support longer-term CAR expression. A seminal study by Smith et al. used synthetic poly (β-amino ester) (PBAE) nanoparticles loaded with plasmid DNA encoding CD19-specific CAR to program circulating T cells in mice. It proved that polymeric DNA nanocarriers can mediate clinically relevant anti-tumour responses without ex vivo manipulation [141]. Mechanistically, these cationic polymers electrostatically complex with negatively charged DNA, protect it from degradation, and facilitate endosomal escape and nuclear entry. Subsequent work and recent reviews have highlighted PBAE and related biodegradable polymers as promising vectors for in vivo immune-cell reprogramming, given their tuneable chemistry, relatively low cost, and ability to co-deliver multiple genetic elements [137]. However, polymeric systems face several limitations. DNA requires nuclear access, which can limit transfection efficiency compared with mRNA-based LNPs. When combined with transposase systems, carries a theoretical risk of insertional mutagenesis. In addition, formulation reproducibility and in vivo biodistribution can vary between batches [142]. Thus, while polymeric DNA nanocarriers offer a flexible, non-viral platform with promising preclinical efficacy, their translation will depend on improved control over targeting, integration, and long-term safety.

### 6.3. Viral Vectors

Viral vectors provided the earliest and most robust demonstrations that CAR-T cells can be generated directly inside the host. Recent work by Nicolai et al. described VivoVec, a T-cell-targeted lentiviral platform designed for in vivo CAR-T generation in non-human primates. The vector displays a multi-domain fusion ligand comprising CD80 and CD58 to engage CD3 and costimulatory receptors on T cells. A single infusion of VivoVec encoding a CD20 CAR generated substantial frequencies of CAR^+^ T cells in blood and secondary lymphoid organs, leading to deep and sustained B cell depletion without the need for lymphodepleting chemotherapy [143]. In parallel, AAV-based systems have also been explored for the generation of in vivo CAR platform. AAV-based systems have similarly shown efficacy in generating functional CAR-T cells in vivo, leading to successful tumour regression [144,145].

These viral approaches offer very high transduction efficiency and long-term expression, supported by mature GMP manufacturing pipelines. However, both platforms face important challenges, including high production cost, pre-existing anti-vector immunity that can impair efficacy or prevent re-dosing, and, for lentivirus, a residual risk of insertional mutagenesis. These constraints are driving the rapid development of non-viral and hybrid systems that aim to retain the potency of viral vectors while improving safety and scalability.

### 6.4. Bioinstructive Implantable Scaffolds

Beyond systemic nanoparticles and viral vectors, bioinstructive implantable scaffolds offer a localized strategy to generate and release CAR-T cells directly within the patient. In a key study, Agarwalla et al. introduced MASTER (Multifunctional Alginate Scaffold for T-cell Engineering and Release), an alginate-based, antibody- and cytokine-functionalized scaffold, in which autologous PBMCs were briefly mixed ex vivo with CAR-encoding viral particles and loaded into MASTER, which was then implanted subcutaneously. Within hours, T cells were activated, transduced, and expanded inside the scaffold and subsequently released into circulation as CAR-T cells. In murine lymphoma models, MASTER-generated CAR-T cells showed superior engraftment, a less exhausted, more stem-like phenotype, and improved tumour control compared with conventionally manufactured CAR-T cells while reducing the overall production timeline to roughly one day [146].

Subsequent work has extended the scaffold concept to acellular systems that recruit and program host T cells directly in situ. Dandia et al. reported a three-dimensional, cell-free scaffold implanted adjacent to solid tumours, loaded with viral vectors and immunostimulatory materials, which locally attracted endogenous T cells, mediated CAR gene transfer, and promoted their expansion within the tumour bed, resulting in marked tumour growth inhibition in preclinical models [146]. Similarly, Pandit et al. developed “Drydux,” a macroporous biomaterial “CAR-T factory” that can be implanted near solid tumours to generate and release tumour-specific CAR-T cells in situ [147].

Bioinstructive scaffolds thus offer several conceptual advantages. They favour early memory phenotypes by minimizing prolonged ex vivo culture and concentrate CAR-T activity at or near tumour sites, which may lower systemic toxicity and improve solid tumour responses. At the same time, they introduce new challenges, including the requirement for surgical or interventional delivery, added complexity in device-level manufacturing and regulation as combination products. Rather than replacing systemic in vivo CAR-T platforms, scaffold-based in situ programming is likely to serve as a complementary strategy, illustrating how the convergence of bioinstructive materials and gene delivery technologies can shape the future of CAR-T manufacturing and clinical translation.

## 7. Conclusions and Perspectives

Over the past decades, CAR technology has evolved into a foundational platform in cancer immunotherapy. By redirecting immune cell specificity through antibody-derived scFvs, CAR-engineered lymphocytes have produced transformative clinical outcomes in relapsed or refractory haematological malignancies. The approval of CD19-targeted products, such as tisagenlecleucel and axicabtagene ciloleucel, has validated this therapeutic concept and paved the way for rapid innovation [4,5]. Despite these achievements, conventional CAR-T therapies remain constrained by manufacturing bottlenecks, high cost, variable product quality, and limited efficacy in solid tumours.

Manufacturing is central to these limitations. The autologous model requires individualized leukapheresis, ex vivo activation, gene transfer, and expansion. These steps are logistically complex, costly, and difficult to scale. Product quality is further influenced by patient-intrinsic factors such as lymphopenia, prior chemotherapy, or immune exhaustion. To address these challenges, manufacturing protocols are evolving rapidly. Automated and closed systems such as the CliniMACS Prodigy^®^, Lonza Cocoon^®^, and G-Rex^®^ now enable GMP-compliant, standardized production with reduced operator input and lower contamination risk [61,63]. Improvements in cytokine support, starting cell phenotype, and activation conditions have further refined product consistency. In parallel, ultrarapid manufacturing platforms, including UF-CAR, FasT CAR-T, and PACK-IT, have reduced production time from weeks to days while preserving early-memory phenotypes and functional potency [64,66,67]. Together, these developments signal a shift toward rapid, decentralized, and more accessible manufacturing models.

Beyond conventional αβ T cells, a new generation of CAR platforms harnesses unconventional immune populations such as γδ T, iNKT, MAIT, and NK cells. These subsets offer intrinsic advantages, including MHC-independent recognition, reduced risk of GVHD, natural tissue tropism, and robust baseline cytotoxicity [98,104,111]. Although most remain at preclinical or early clinical stages, advances in isolation, expansion, and transduction, such as MR1-tetramer sorting, feeder-cell systems, and optimized lentiviral pseudotypes, are accelerating their translational readiness [127].

Another transformative development is in vivo CAR-T engineering, where circulating T cells are genetically programmed inside the patient using lipid nanoparticles or polymeric carriers that deliver CAR-encoding mRNA or DNA [136,140]. This approach bypasses ex vivo manipulation and has the potential to reduce cost and broaden global access. Preclinical studies have shown efficient generation of functional CAR-T cells in vivo with robust activity in models of B cell depletion and cardiac fibrosis [136,148], and early-phase clinical evaluation is underway.

Despite these technological advances, solid tumours remain the most formidable barrier. Antigen heterogeneity, physical stromal barriers, and a highly immunosuppressive TME collectively limit CAR-T cell infiltration, persistence and effector function. To overcome these obstacles, several complementary engineering strategies are under active investigation. Metabolic reprogramming, such as overexpressing the mitochondrial regulator PGC1α or disrupting *REGNASE-1*, aims to enhance oxidative metabolism and nutrient utilisation to improve T cell fitness within the nutrient-depleted TME. Checkpoint modulation, including PD-1 knockout or PD-1/CD28 switch-receptor designs, can restore T cell activation in the presence of inhibitory ligands. Enzymatic strategies, such as ADA1-mediated degradation of adenosine, counteract metabolite-driven suppression in the tumour niche [149,150,151]. In parallel, nanocarrier-delivered siRNA targeting PD-L1 or TGF-β pathways, together with cytokine-armoured or matrix-remodelling CAR-T constructs, enhance tumour infiltration and functional resilience [152,153]. These complementary approaches demonstrate how manufacturing innovation and cellular engineering are converging to expand CAR-T applications beyond haematologic cancers.

While these innovations broaden the therapeutic landscape, several cross-cutting challenges must be resolved to ensure scalable, standardized, and clinically robust CAR-based therapies. Large-scale expansion of rare populations such as MAIT and iNKT cells is technically demanding. Robust, harmonized potency assays and real-time quality control will be essential to ensure inter-site consistency as automated systems become more prevalent. Gene-transfer platforms, such as viral, non-viral, and CRISPR-based, must continue to balance efficiency, cost, scalability, and genomic safety. Meanwhile, combinatorial engineering strategies, including logic-gated CARs, suicide switches, and cytokine armouring, are being refined to enhance precision and mitigate toxicity [44,154]. Integration of synthetic biology, single-cell analytics, and machine-learning–supported optimisation is expected to accelerate innovation across the CAR manufacturing ecosystem.

In conclusion, the CAR field is transitioning from custom-built, labour-intensive therapies toward programmable, scalable, and globally deployable platforms. The convergence of manufacturing innovation, novel immune cell sources, and in vivo CAR engineering is poised to broaden the reach of CAR-based immunotherapy across both haematologic and solid tumours. As these technologies mature, the establishment of harmonized regulatory frameworks, standardized potency metrics, and international quality guidelines will be critical to translating their full clinical and societal impact.

## Figures and Tables

**Table 1:** Overview of representative CAR-T cell products on the market for cancers.

Target Antigen	Diagnosis	Company/Institution	Year	Approval
CD19	B-ALL DLBCL	Novartis	2017 (FDA) 2018 (EMA)	FDA/EMA-approved Kymriah^®^ (Tisagenlecleucel)
CD19	LBCL DLBCL FL	Kite Pharma	2017 (FDA) 2018 (EMA) 2021 (NMPA)	FDA/EMA/NMPA-approved Yescarta^®^ (Axicabtagene Ciloleucel)
CD19	LBCL DLBCL	Juno Therapeutics	2021 (FDA) 2022 (EMA)	FDA/EMA-approved Breyanzi^®^ (Lisocabtagene Maraleucel)
CD19	MCL	Kite Pharma	2020 (FDA, EMA)	FDA/EMA-approved Tecartus^®^ (Brexucabtagene Autoleucel)
CD19	LBCL FL MCL	JW Therapeutics	2021	NMPA-approved Carteyva^®^(Relmacabtagene Autoleucel)
CD19	B-ALL DLBCL	Juventas Cell Therapy/CASI Pharmaceuticals	2021	NMPA-approved Yorwida^®^ (Inaticabtagene Autoleucel)
BCMA	MM	Celgene Corporation	2021 (FDA, EMA)	FDA/EMA-approved Abecma^®^ (Idecabtagene Vicleucel)
BCMA	MM	Janssen Biotech	2022 (FDA, EMA) 2023 (NMPA)	FDA/EMA/NMPA-approved Carvykti^®^ (Ciltacabtagene Autoleucel)
BCMA	r/r MM	IASO Bio/Innovent Biologics	2023	NMPA-approved Fucaso^®^(Equecabtagene Autoleucel)
BCMA	r/r MM	CARsgen Therapeutics	2024	NMPA-approved Zevor-cel^®^ (Zevorcabtagene Autoleucel)

**Table 2:** Clinical Trials of Allogeneic CAR-Modified Innate and Unconventional Immune Cell Therapies.

Immune Cell Subtype	Target	No.	Company/Institution	Phase	Date
γδ T Cells	CD20	NCT04735471 NCT04911478	Adicet Therapeutics	Phase I/ Long term observational	2021–2025 2022–2039
	NKG2DL	NCT04107142	CytoMed Therapeutics Pte Ltd.	Phase I	2019–2021
	HLA-G	NCT06150885	Ever Supreme Biotechnology Co., Ltd.	Phase I/II	2024–2027
iNKT Cells	CD19	NCT03774654	Carlos Ramos, Baylor College of Medicine	Phase I	2020–2035
	CD19	NCT05487651	Athenex, Inc.	Phase I	2022–2024
	GD2	NCT03294954	Andras Heczey, Baylor College of Medicine	Phase I	2020–2035
NK Cells	CD19	NCT05020678	Nkarta, Inc.	Phase I	2021–2038
	CD19	NCT03056339	M.D. Anderson Cancer Center	Phase I/II	2017–2023
	CD19	NCT04245722	Fate Therapeutics	Phase I	2020–2023
	HER2	NCT03383978	Johann Wolfgang Goethe University Hospital	Phase I	2017–2026
	PD-L1	NCT04847466	National Cancer Institute (NCI), US	Phase II	2021–2027
	Claudin-6, GPC3, Mesothelin, AXL	NCT05410717	Second Affiliated Hospital of Guangzhou Medical University	Phase I	2022–2036

**Table 3:** Key features of delivery systems enabling in vivo CAR-T engineering.

Platform	Composition	Cargo	Mechanism of Action	Advantages	Limitations
Lipid Nanoparticles	Ionizable lipid + cholesterol + PEG-lipid; may include antibody–lipid conjugate for targeting.	mRNA DNA	Endocytosis → mRNA translation → transient CAR expression	Safe Non-viral, scalable (vaccine-like manufacturing) Subset-specific targeting	Transient expression Innate immune sensing Hepatic accumulation risk
Polymeric Nanoparticles	Biodegradable or cationic polymers (PLGA, PEI, chitosan)	mRNA mcDNA	Uptake by endocytosis → proton-sponge–mediated endosomal escape → cytoplasmic/nuclear delivery	Highly customizable (surface charge, degradation rate). Can co-deliver adjuvants or cytokines.	Lower T cell efficiency Risk of insertional mutagenesis Variable biocompatibility Reproducibility issues
Lentiviral Delivery System	Enveloped RNA virus (HIV-1–derived; VSV-G pseudotyped)	RNA	Reverse transcription → genomic integration → stable CAR expression	Long-term expression Clinically validated High efficiency	Integration risk (mutagenesis) Costly GMP manufacturing Limited redosing
AAV-Based Delivery System	Non-enveloped capsid with single-stranded or self-complementary DNA genome (AAV2, AAV6, AAV9)	DNA	Nuclear entry → episomal persistence (non-integrating) → transient/semi-stable expression	High efficiency Low genotoxic risk	Small cargo capacity (~4.7 kb) Anti-AAV immunity Expensive
Bioinstructive Implantable Scaffold	Biodegradable hydrogel/polymer matrix (alginate, PEG) with viral vectors, antibodies, cytokines	Viral or mRNA CAR constructs	PBMCs infiltrate scaffold → in situ transduction & expansion → systemic CAR-T release	Localized CAR induction Controlled cytokine milieu Suitable for solid tumours	Invasive implantation Scalability Preclinical stage

## Data Availability

No new data were created or analyzed in this study.
